# Health status of children left behind in rural areas of Sichuan Province of China: a cross-sectional study

**DOI:** 10.1186/s12914-019-0191-9

**Published:** 2019-01-28

**Authors:** Daisheng Tang, Weng I. Choi, Liyuan Deng, Ying Bian, Hao Hu

**Affiliations:** 1 School of Economics and Management, Beijing Jiaotong University, Haidian, Beijing, 100044 China; 2 State Key Laboratory of Quality Research in Chinese Medicine, Institute of Chinese Medical Sciences, University of Macau, Macao, China; 3 School of Law, Chengdu University of Technology, Chengdu, 610000 Sichuan China

**Keywords:** Left-behind children, China, Physical health, Mental health, Household registration

## Abstract

**Background:**

In recent decades, many workers from rural areas in China migrated to urban cities in search of a better livelihood. Due to the household registration policy and other financial barriers, more than 40 million of children were left behind in their rural home by their migrated parents in 2015. In this cross-sectional study, we aimed to investigate the influence of being left behind on these children’s physical and mental health.

**Methods:**

A self-administered questionnaire was completed by participants about their demographic background and health status. Chi-square Test was conducted to investigate the influence.

**Results:**

A total of 1662 participants responded and completed all the questions in the questionnaire. Significant differences existed between left-behind children group and non-left-behind children group on several health issues such as not going to school due to sickness (*p* = 0.080), completeness of the vaccination scheme (*p* = 0.036) and feeling of loneliness (*p* = 0.039). However, regarding symptoms like fever, cough or respiratory difficulties, diarrhea and twitch, as well as mental health problems like unhappiness and insomnia, no significant difference was found. Gender difference was also demonstrated showing that girls were more vulnerable than boys to certain symptoms and emotional problems.

**Conclusion:**

This study indicated that both being left-behind and gender had an impact on the children’s health. It is necessary to further reform the household registration system to improve rights of equal access to employment, education and health resources for workers and their children from rural areas in China.

**Electronic supplementary material:**

The online version of this article (10.1186/s12914-019-0191-9) contains supplementary material, which is available to authorized users.

## Background

Under the promulgation of economic reform policy in 1978, China has transformed from a planned system to a market-oriented economy, resulted in an accelerated process of industrialization and economic growth. In order to fulfill the tremendous labor demand, governmental policies were issued to permit and even encourage the labor migration from rural to urban areas. Meanwhile, rapid development in agricultural technology reduced the labor demand in rural areas. Consequently, a large-scale internal rural-to-urban labor migration was resulted. Migrant workers were defined as the population not working and living in their registered residential place for six months or longer. According to the 2010 Population Census of The People’s Republic of China, the number of migrant works numbered at more than 170 million [1] as compared to 22 million in 1990 [2]. Until now, the increasing trend is still accelerating due to the uneven economic development between urban and rural areas. This dramatic increase in productivity explained why China’s Gross Domestic Product (GDP) has remarkably risen from 19 billion United Stated Dollar (USD) in 1980 to 12 trillion USD in 2017 [3, 4]. However, this economic prosperity was also associated with various social issues such as the emergence of left-behind children, raising general awareness within the society. Household registration (*hukou*) system which was established officially in 1958 is one of the underlying causes for the left-behind children phenomenon. Under this system, each Chinese citizen was registered according to his/her permanent residential area, based on which the residents were consequently classified into rural or urban categories. This classification led to unequal accessibility to governmental resources between rural and urban residents, including education, healthcare and retirement pension etc. The residents in rural areas usually had less access to employment, education and healthcare resources. Despite the relaxation of this household registration policy over decades, the overall situation was still not favorable for the migrants relocating their families to urban area. It is particularly difficult for the migrants’ children to move in order to receive education at urban public schools due to the limits of household registration system. Therefore, a large population of children and elderly were left behind in their residential places.

The affected children being left behind has been booming due to the unprecedented internal migration. Compared with the data in 2000, more than 40 million children were left behind in rural home in 2015, accounting for almost 15% of total children population [5]. This increasing group represents an important segment of youth population and warrants public concerns over the profound effects. On one hand, better employment and higher income can improve the financial situation of the rural family [6, 7]. With improved financial income, the children’s living condition, nutrition and health status could be improved [8]. Apart from materialistic benefits, migrants may widen their horizon and gain inspiration through their work life in urban areas [9]. Subsequently, left-behind children might benefit from the exposure to new environments and perspectives. Taking education as an example, migrated parents might become more aware of the importance of the education quality for their children [10]. However, being separated from the parents could also be problematic not only to the development of a parental relationship, but also to the children’s health, academic result and performance in school, and sense of security. As reported previously about school performance, left-behind children had poorer academic result than those whose parents did not migrate [11].

It has been well reported that family structure and care is always one of the dominant factors affecting children’s development [12–15]. Lack of parental support and supervision could bring significant effects on children which prompted attention of scholars from different fields. Among these, health status and health behaviors are the most concerning, influential and lifelong developmental outcome, and the evidence is complex. Comparing to non-left-behind children, some studies indicated that left-behind children had poor health and nutritional status (e.g. height, weight, dietary intake etc.) [16–18]. Due to weak parent-child bonding, left-behind children were more likely to experience unhappiness, depression, loneliness, anxiety and to consider leaving home prematurely among Asian populations [19–22]. Studies conducted in Asian countries and Caribbean area reported that left-behind children were more likely to have unhealthy behaviors, such as physical inactivity, internet addiction, smoking and being overweight [19, 23–27]. Moreover, lower coverage rate of vaccination, lower medical services utilization, and lower health expenditure were observed among left-behind children [17, 28]. On the other hand, some studies showed no differences in aspects of psychological and behavioral problems between left-behind children and comparison group [29, 30]. It was even found that left-behind children had a higher height-for-age z-scores among Central American and Central Asian populations [31, 32].

Global studies suggested that parents played a key role in children development through their communication, support and supervision. With such influential impacts, scholars from various field have investigated impacts of family separation, such as divorce or death of one of the parents, on multidimensional developmental outcomes of children, including health, education and behaviors etc. [33]. Among these, a small portion of studies examined the influence of migration-induced parental absence [27]. Due to the late emergence of left-behind children in China, most studies mainly examined the issues in western countries, such as Mexico and Philippines, in where left-behind children was a prominent phenomenon.

Previous studies conducted in China have researched about the impacts of parental migration on education and psychological health. At present, little research focuses on the impact on physical health of left-behind children. There is only limited research on the physical health of the left-behind children which only reported about whether the participants feel sick or not during a given period of time. The data about the impact on specific health signs and symptoms, utilization of medical services, coverage and concepts of vaccination remains scarce. In addition, mixed results were yielded from the literature concerning the impact on the left-behind children. As the current empirical result in China is limited and inconclusive, further studies are warranted to further determine whether there is any difference in the developmental outcomes of children by the pattern of parental migration.

In this study, we aimed to investigate whether there is an impact of parental migration among the junior high school students in Sichuan, through the comparison with those living in the same area and whose parents did not undergo migration. The two primary research questions were:

1. Is there any difference between left-behind children and comparison group regarding their physical health (e.g. symptoms experienced and other related health behaviors)?
2. Is there any difference between left-behind children and comparison group regarding their mental health (e.g. emotional problems experienced and other related health behaviors)?

## Methods

### Study population and selection procedure

This study was reviewed and approved by the Ethics Committee of Beijing Jiaotong University (Project No. 14BRK027). A cross sectional study was conducted from April 20 to 22,2017 in Sichuan, one of the most populated province in China, with more than 82 million residents in 2016 [1]. Sichuan is a province in southwest China, covering 486,000 km^2^ and being featured by the amount of labour exporting each year. According to the data from 2015 China consensus, more than 12 million residents involved in inter-provincial migration [2]. With the internal rural-to-urban migration, it has been estimated that the migration population almost takes up one-third of the provincial population.

In this study the target participant were junior high school students (aged 12–15 years). The participants were recruited by multi-stage sampling method as followed: 1) In order to obtain a proper sample size of eligible participants, cluster sampling approach was used for selecting areas with more rural-to-urban migrants. Six cities or autonomous prefectures were chosen in this stage, including Dazhou city, Bazhong city and Nanchong city in North- Eastern Sichuan, Neijiang city and Yibin city in Southern Sichuan, and Liangshan Autonomous prefectures in North-Western Sichuan; 2) The main selection criteria of the sampling counties are counties with large population of migrants and so left-behind children. Also, the selected counties should be geographically representative for Sichuan Province. As a result, four counties (Tongjiang County, Zizhong County, Gao County, Yuexi County), one district (Dachuan District) and one city (Liangzhong city) were purposively selected based on the mentioned criteria mentioned above; 3) Based on the population size of the selected area, the sample size were 800, 1000 and 1200 participants for areas with small (Yuexi County), middle (Tongjiang County) and large (Gao County, Zizhong County, Liangzhong city and Dachuan District) population scales respectively; 4) For left-behind children related research, National Health and Family Planning Commission of PRC has determined the ratio of left-behind children and non-left-behind children as 2:1. Therefore, participants were recruited through quota sampling with the stated ratio. For example, apart from Yuexi County, 200 left-behind children and 100 non left-behind children were selected in each county or city. In total, 1160 left-behind children and 580 non left-behind children were invited for this study; 5) The exclusion criteria for sampling population include children with divorced parents, either father or mother of the children passed away, or the children were orphans.

In this study, with the assistance of the Sichuan Population Association, the first contact was made with the school teachers. Then, the school teachers, upon agreeing to help, distributed the research invitations to the children’s legal guardians to seek their consent of participating in this study. The legal guardians in this study were parents for non-left-behind children and grandparents (or other close family relatives such as uncles, etc. who is taking care of the children) for left-behind children. The survey was conducted when all the written consent from the legal guardian was received. The participants were informed to bring certain documents (e.g household register, vaccination record and health check reports within recent 3 months etc.) with them on the date of filling questionnaire to allow the collection of essential information for the purpose of this study. A self-administrated questionnaire was provided to each participant. All the participants were informed of the objective of this study and assurance of the confidentiality of their personal information before filling the questionnaire. Participants were aware of their right to withdraw from this study any time. In order to assure the validity of data, participants were required to keep certain distance away from each other when they were filling the questionnaire. This helped to avoid discussion between participants and keep the confidentiality of their personal information. Due to some participants’ withdrawal and the exclusion of ineligible questionnaires due to substantial missing data, 1662 participants were included in the analysis.

### Data collection and measurement

In the pilot study, it was found that left-behind children generally felt uncomfortable to fill the questionnaire with standardized scale. Therefore, the research team developed the survey questionnaire according to the field work conducted by the research team in Sichuan Province previously. The measurement items were mainly adopted from the findings from qualitative interviews with local teachers and medical staffs who had taken care of the left-behind children before. While the measurement items are simple questions, they reflect the realistic life of the left-behind children and can be easily comprehended.

The final questionnaire mainly consisted of three main parts: demographic characteristic, physical health and mental health (see Additional file 1). Participants were asked about their age, gender, rural household registration (Yes or No), both parents migrating for employment more than 6 months (Yes or No), and maternal and paternal education level (illiterate, primary school, junior high school, high school, college, university or above). Regarding to left-behind issues, ‘left-behind children’ in this study were defined by the following criteria: 1) children under 16 years old; 2) with rural household registration; 3) being left behind in the original residence for 6 months or longer due to parents’ migration to other place for employment and therefore not being able to live together. For those children with one parent migrating or none of the parents migrating for employment, they were classified as non left-behind children.

Physical health status was measured by the occurrence of several symptoms (fever, cough or respiratory difficulties, diarrhea and twitch) in the past two weeks. Health behaviors were also investigated, for example, visiting healthcare institute due to sickness in the past two weeks and the type of healthcare institute visited (private clinic / private hospital, village clinic, township hospitals, and county hospitals and above). In addition, the participants were asked to report the frequency of not going to school due to sickness and taking sick leaves in the past 12 months, as well as having the vaccination document and completeness of the national vaccination scheme.

Similarly, the assessment of mental health status included the measurement of frequency (never, seldom, sometimes, often, always) of several emotional problems in the past 6 months, such as unhappiness due to the study stress or the issues related to the academic result, insomnia due to worrying and loneliness. Besides, question like ‘Have you had the idea of running away from home in the past 6 months (1=Never thought about it; 2=Have thought about it; 3=Tried to run away but failed; 4=Ran away from home)?’ and ‘Have you ever stopped the daily activities for 2 consecutive weeks or even longer time due to depression or hopelessness? (1=Yes; 2=No)’ were asked to identify the participants’ situation regarding to this aspect.

### Data analysis

In order to improve the quality of the data, trained interviewer checked the submitted questionnaire for any missing or ambiguous data. Once it was completed, the participants signed the questionnaire and left the contact number for possible future contact. Also, double entry of questionnaires was performed by different researchers to minimize data error.

Data were input into and analyzed by SPSSS statistical software program. Chi square test was applied to examine the between-group differences of 1) Left-behind group and non-left-behind group; 2) Male and female in left-behind children group; among the physical health and mental health indicators.

## Results

### Demographic information

The summary of demographic characteristic is shown in Table 1. Among 1662 participants, 853 (50.2%) were boys and 827 were girls, giving almost equal proportion of male and female. Among the participants, 1115 students were left behind by parents or either father or mother with 547 students in non-left-behind group. Many of the fathers (56.1%) and mothers (49.0%) were educated at the level of junior high school or above while 32.7% of the fathers and 36.9% of the mothers had primary school education.

**Table 1:** Demographic variables of the participants

Variables	No of students	Percentage (%)
Gender		
Male	835	50.2
Female	827	49.8
Left-behind Child		
Yes	1115	67.1
No	547	32.9
Paternal Education		
Illiterate	59	3.5
Primary School	544	32.7
Junior High School	855	51.4
High School	176	10.6
College	19	1.1
University or above	3	0.2
Maternal Education		
Illiterate	173	10.4
Primary School	614	36.9
Junior High School	734	44.2
High School	120	7.2
College	12	0.7
University or above	2	0.1

### Physical health status

In general, apart from cough or respiratory difficulties, left-behind children had a higher prevalence of all the symptoms as shown in Table 2. The difference ranged from 0.7 to 3%. In spite of the higher prevalence of these symptoms among the left-behind children group, proportionally more non-left-behind children (49.7%) visited healthcare institute in the past 2 weeks. On the choice of visited healthcare institute, left-behind children mostly chose township hospital (36.6%) while non-left-behind children were more likely to seek medical care at private clinic/private hospital (34.8%). Overall, there was no significant difference in all these categories. Both majority of the left-behind children (70.4%) and no- left-behind children (71.5%) had not taken any sick leave from school in the past 12 months. More non-left-behind children took sick leave once (20.2%) while more left-behind children took sick leaves twice or more (6.3%). Significant difference was found between these two groups with regards to the frequency of not going to school due to sickness (*p* = 0.080). Similarly, more non-left-behind children did not get sick in the past 12 months while more left-behind children got sick (no matter take leave or not). The difference was significant (*p* = 0.011). While 51.5% of non-left-behind children had the vaccination document, which the portion was more than left-behind children, the difference was not significant. Higher percentage of non-left-behind children (44.2%) completed free vaccination provided by government than left-behind children (38.7%). Regarding to the health behaviors, significant difference was found between the two groups (*p* = 0.036).

**Table 2:** Physical health variables of the participants between left-behind children group and non left-behind children group

Physical health	Variables	Left-Behind Children	Non Left-Behind Children	*p*-value
Health status	Fever			
	Yes	270 (24.2)	115 (21.0)	0.147
	No	845 (75.8)	432 (79.0)	
	Cough or respiratory difficulties			
	Yes	394 (35.4)	201 (36.7)	0.582
	No	720 (64.6)	346 (63.3)	
	Diarrhea			
	Yes	356 (32.0)	161 (29.4)	0.291
	No	757 (68.0)	386 (70.6)	
	Twitch			
	Yes	22 (2.0)	7 (1.3)	0.309
	No	1092 (98.0)	540 (98.7)	
Health behaviour	Visit healthcare institute due to sickness			
	Yes	297 (47.1)	151 (49.7)	0.469
	No	333 (52.9)	153 (50.3)	
	Type of healthcare institute visited			
	Private clinic/private hospital	89 (30.2)	54 (34.8)	0.520
	Village clinic	59 (20.0)	35 (22.6)	
	Township hospitals	108 (36.6)	47 (30.3)	
	County hospitals and above	39 (13.2)	19 (12.3)	
	Not going to school due to sickness			
	Never	781 (70.4)	389 (71.5)	0.080*
	1 time	207 (18.6)	110 (20.2)	
	2 times	70 (6.3)	28 (5.1)	
	3 times	38 (3.4)	7 (1.3)	
	4 times or above	14 (1.3)	10 (1.8)	
	Take sick leave from school due to sickness			
	Did not get sick	337 (30.3)	204 (37.7)	0.011**
	Sick but did not take leave	532 (47.9)	233 (43.1)	
	Sick and take leave	242 (21.8)	104 (19.2)	
	Having the vaccination document			
	Yes	533 (47.8)	281 (51.5)	0.371
	No	92 (8.3)	41 (7.5)	
	Do not know	490 (43.9)	224 (41.0)	
	Complete the corresponding vaccination scheme for your age			
	Yes	432 (38.7)	241 (44.2)	0.036**
	No	117 (10.5)	64 (11.7)	
	Do not know	566 (50.8)	240 (44.0)	

* *p* < 0.1; **p < 0.05; ***p < 0.01;

In Table 3, the physical health variables between male and female among left-behind children were analyzed. The prevalence of fever, cough or respiratory difficulties and diarrhea was higher among girls (25.5, 39.1 and 34.7% respectively) than boys (23.0, 31.7 and 29.3% respectively). On the contrary, the prevalence of twitch was higher among boys (2.1%) than girls (1.8%). Significant difference was found not only in cough or respiratory difficulties (*p* = 0.010), but also in the category of diarrhea (*p* = 0.053). Both half of the boys (44.7%) and girls (49.3%) who reported symptoms in the past 2 weeks visited healthcare institutes. Regarding to the choice of healthcare institutes, most boys and girls chose township hospitals (35.1 and 37.8%), in which the choice of left-behind boys was different from all boys (the majority chose private clinics/private hospital). In addition, concerning to the behavior of taking sick leaves from school, most of the boys (43.2%) and girls (52.6%) reported they were sick in the past 12 months but did not take sick leave while for those being sick and took leaves, more boys (23.1%) took sick leaves than girls (20.4%), and this difference in this category was significant (*p* = 0.006). More girls (48.0%) had the vaccination document than boys (47.6%) while more boys (40.4%) completed the vaccination scheme than girls (37.1%). However, these differences were not significant.

**Table 3:** Physical Health Variables between male and female among left-behind children

Physical health	Variables	Male	Female	P
Health status	Fever			
	Yes	130 (23.0)	140 (25.5)	0.341
	No	435 (77.0)	410 (74.5)	
	Cough or respiratory difficulties			
	Yes	179 (31.7)	215 (39.1)	0.010**
	No	385 (68.3)	335 (60.9)	
	Diarrhea			
	Yes	165 (29.3)	191 (34.7)	0.053*
	No	398 (70.7)	359 (65.3)	
	Twitch			
	Yes	12 (2.1)	10 (1.8)	0.711
	No	552 (97.9)	540 (98.2)	
Health behaviour	Visit healthcare institute due to sickness			
	Yes	132 (44.7)	165 (49.3)	0.258
	No	163 (55.3)	170 (50.7)	
	Type of healthcare institute visited			
	Private clinic/private hospital	45 (34.4)	44 (26.8)	0.527
	Village clinic	23 (17.6)	36 (22.0)	
	Township hospitals	46 (35.1)	62 (37.8)	
	County hospitals and above	17 (13.0)	22 (13.4)	
	Not going to school due to sickness			
	Never	391 (69.4)	390 (71.3)	0.718
	1 time	104 (18.5)	103 (18.8)	
	2 times	37 (6.6)	33 (6.0)	
	3 times	22 (3.9)	16 (2.9)	
	4 times or above	9 (1.6)	5 (0.9)	
	Take sick leave from school due to sickness			
	Did not get sick	189 (33.6)	148 (27.0)	0.006***
	Sick but did not take leave	243 (43.2)	289 (52.6)	
	Sick and take leave	130 (23.1)	112 (20.4)	
	Having the vaccination document			
	Yes	269 (47.6)	264 (48.0)	0.913
	No	45 (8.0)	47 (8.5)	
	Do not know	251 (44.4)	239 (43.5)	
	Complete the corresponding vaccination scheme for your age			
	Yes	228 (40.4)	204 (37.1)	0.503
	No	56 (9.9)	61 (11.1)	
	Do not know	281 (49.7)	285 (51.8)	

* *p* < 0.1; **p < 0.05; ***p < 0.01;

### Mental health status

Table 4 illustrated the comparison of unhappiness, insomnia, loneliness, ideation of running away from home and stopping the daily activities for 2 consecutive weeks due to depression or hopelessness between left-behind children and non-left-behind children. For unhappiness, insomnia and loneliness, the situation was alike as higher proportion of left-behind children reported that they had not experienced these emotional problems in the past 2 weeks than non left-behind children. Surprisingly, the differences were quite small ranging from 0.5 to 0.9%. On the other hand, the majority of both left-behind children (72.1%) and non left-behind children (76.2%) reported that they had never thought of running away from home, but non-left-behind children were more in portion comparatively. Moreover, compared to non-left-behind children, more left-behind children stopped the daily activities for 2 consecutive weeks or even longer time due to depression or hopelessness. Overall, among these mental health variables, the chi-square tests showed that there were only significant differences between the two groups in terms of loneliness (*p* = 0.039).

**Table 4:** Mental health variables of the participants between left-behind children group and non left-behind children group

Variables	Left-Behind Children	Non Left-Behind Children	*p*-value
Unhappiness due to the study stress or the issues related to the academic result			
Never	142 (12.7)	67 (12.2)	0.990
Seldom	242 (21.7)	114 (20.8)	
Sometimes	502 (45.0)	252 (46.1)	
Often	133 (11.9)	66 (12.1)	
Always	96 (8.6)	48 (8.8)	
Insomnia due to worrying			
Never	438 (39.3)	210 (38.4)	0.889
Seldom	326 (29.2)	163 (29.8)	
Sometimes	268 (24.0)	126 (23.0)	
Often	55 (4.9)	31 (5.7)	
Always	28 (2.5)	17 (3.1)	
Loneliness			
Never	403 (39.3)	231 (38.4)	0.039**
Seldom	255 (29.2)	135 (29.8)	
Sometimes	292 (24.0)	115 (23.0)	
Often	130 (4.9)	53 (5.7)	
Always	35 (2.5)	13 (3.1)	
Having the idea of running away from home			
Never thought	804 (72.1)	417 (76.2)	0.289
Have thought about it	291 (26.1)	119 (21.8)	
Tried to run away but failed	13 (1.2)	7 (1.3)	
Ran away from home	7 (0.6)	4 (0.7)	
Stopping the daily activities for 2 consecutive weeks or even longer time due to depression or hopelessness			
Yes	148 (13.3)	68 (12.4)	0.631
No	967 (86.7)	479 (87.6)	

* *p* < 0.1; **p < 0.05; ***p < 0.01;

Most of the boys (38.9%) and girls (51.3%) among left-behind children sometimes felt unhappy in the last 6 months, in which the prevalence of girls was much higher. Meanwhile, more boys (44.1 and 40.7% respectively) did not encounter insomnia or loneliness than girls (34.4 and 31.5% respectively). In these three categories, the prevalence of girls was higher than boys and significant differences were observed (*p* = 0.000, *p* = 0.013 and *p* = 0.000 respectively). Furthermore, compared with boys (69.6%), more girls (74.4%) never thought of running away from home while more boys thought of running away, tried to run away but failed or had successfully ran away before. When asked about whether they had stopped daily activities for 2 consecutive weeks or even longer time due to depression or hopelessness, more boys (13.6%) reported to have this problem than girls (12.9%). The differences in these two questions did not achieve the significant level (see Table 5).

**Table 5:** Mental health variables between male and female among left-behind children

Variables	Male	Female	*p*-value
Unhappiness due to the study stress or the issues related to the academic result			
Never	88 (15.6)	54 (9.8)	0.000***
Seldom	144 (25.5)	98 (17.8)	
Sometimes	220 (38.9)	282 (51.3)	
Often	59 (10.4)	74 (13.5)	
Always	54 (9.6)	42 (7.6)	
Insomnia due to worrying			
Never	249 (44.1)	189 (34.4)	0.013**
Seldom	158 (28.0)	168 (30.5)	
Sometimes	119 (21.1)	149 (27.1)	
Often	24 (4.2)	31 (5.6)	
Always	15 (2.7)	13 (2.4)	
Loneliness			
Never	230 (40.7)	173 (31.5)	0.000***
Seldom	140 (24.8)	115 (20.9)	
Sometimes	117 (20.7)	175 (31.8)	
Often	60 (10.6)	70 (12.7)	
Always	18 (3.2)	17 (3.1)	
Having the idea of running away from home			
Never thought	393 (69.6)	411 (74.7)	0.269
Have thought about it	160 (28.3)	131 (23.8)	
Tried to run away but failed	8 (1.4)	5 (0.9)	
Ran away from home	4 (0.7)	3 (0.5)	
Stopping the daily activities for 2 consecutive weeks or even longer time due to depression or hopelessness			
Yes	77 (13.6)	71 (12.9)	0.732
No	488 (86.4)	479 (87.1)	

* *p* < 0.1; **p < 0.05; ***p < 0.01;

## Discussion

The present cross-sectional study revealed that there was significant difference between the left-behind children and non-left-behind children in terms of health-related behaviors (e.g. the frequency of absence at school due to sickness and the completeness of vaccination scheme) and emotional problems (e.g. loneliness) in the community in Sichuan Province. More left-behind children did not go to school due to sickness and took the sick leave in the past 12 months. Regarding to the frequency of absence in school, more portion of left-behind children reported with ‘2 times’, ‘3 times’ and ‘4 times’. Although the majority of both left-behind children and non-left-behind children took sick leave for one day or less, there were higher percentage of left-behind children took sick leave for more days. This finding presented that left-behind children were more likely to be absent in school and needed a longer leave from school due to sickness. In addition, regarding the completeness of national vaccination scheme, fewer left-behind children received all the free vaccines provided by government. Both difference in two groups are significant and consistent with the previous researches carried out in different countries [17, 28]. Such differences could be potentially explained by the impact of household registration system. First, because of the access to education resource is highly restricted to household registration, children in rural areas cannot go to urban with their parents, which may contribute to the absence of parental care. Usually, the left-behind children were taken care by other family members when their parents migrated to cities and in most cases, the caregivers were their grandparents. Due to the differences in the education level, health awareness and traditional lifestyle, left-behind children may not receive proper treatment when they are ill or preventive measure such as vaccination for health protection. This could result in longer recovery time, more frequent occurrence of illness, and lower coverage of vaccination compared to non-left-behind children. Second, because healthcare resources are also related to household registration, left-behind children receive less health service from public health systems. It should be noted that in the two questions related to vaccination, around 40 to 50% of participants did not know whether they had the vaccination document and had received all the free vaccines. In a summary, the economic, education and healthcare embedded with household registration system obviously affected the health status and behaviors of left-behind children.

However, more left-behind children never felt lonely in the past 6 months, which was not consistent with some previous studies conducted in China [34–36]. Meanwhile, no significant difference has been observed among the categories of physical symptoms, the type of healthcare institute visited and some emotional problems between the two groups. These findings suggested that protective factors potentially existed which prevented the adverse effects of parental migration on left-behind children [37]. Increase in resource accessibility of the children could be one of the potential reason since the migrated parents gain a higher income and so improved the financial situation of their rural household [38]. It implies that change of rights to better employment for migrants from rural areas could bring health benefits to their children left behind.

In left-behind children group, higher prevalence of several physical symptoms (e.g. cough or respiratory difficulties and diarrhea), school attendance (take sick leave from school) and emotional problems (e.g. unhappiness, insomnia and loneliness) was illustrated among female than male. These significant differences are potentially attributed by gender discrimination in rural areas. It is still common to see many parents in rural areas pay more attention on their sons than daughters because girls are thought to be less economically productive for the family. In case of sickness or even hospitalization, compared with boys, girls in rural China received less care requiring less economic spending from family [39]. Consequently, the health inequalities caused by gender differences are still relatively obvious in rural China.

Despite the mentioned limitation, several implications and applications related to left-behind children are worth-noting. This study provides the local findings and most current picture of the provincial situation, which could be treated as baseline data for future research and follow-up. Furthermore, based on the results from this study, compared with non-left-behind children and male, worse health status and health related-behavior were reported among left-behind children and female. Targeting these specific subgroups, three policy suggestions are proposed. First, reform of rights to employment and education for rural labor and their family should be further implemented. As shown in this study, most health issues of left-behind children are rooted from the limits of household registration system. While some improvement of the system has been introduced recently, fundamental changes of the system to provide equal accessibility to public goods is essentially crucial. Second, the unbalance gender issue should also be corrected so that the worse health situation among female left-behind children could be improved. Policy specific to education and health of female children in rural areas should be designed and introduced to decrease gender inequality currently existing in rural areas. Third, health promotion and education program should be developed and adjusted. In these programs, different stakeholders should be addressed in order to provide comprehensive support to those in needs. Specified key message should be delivered to various target groups to maximize the effects. For instance, health education about correct health related knowledge and health seeking behaviors should be given to caregivers which aims to increase their awareness and develop a correct concept.

In the current study, there are also some limitations. The sampling framework was based on the participants selected from relatively small poor rural areas in Sichuan province, though we maximized the representativeness of this study by selecting different areas in the same province. It should be aware during the extrapolation of the findings to all left-behind children in China, since the circumstances vary among different regions in China in terms of socioeconomic background, destination of migration, climate, nutrition and culture etc. Also, as the participants were recruited from school, this might lead to selection bias due to the undercoveage of those who did not enroll or attend in school. Meanwhile, the instrument used in this study was self-administered questionnaire and participants might selectively report or suppress the information required, resulting in reporting bias. Future application of mature standardized scale measuring physical and mental health is needed. In addition, the cross-sectional study design does not allow for causality analysis between the physical and mental health outcomes and parental migration. The leading cause of the observed differences was not identified in this study as well. Therefore, further longitudinal analysis is needed to determine the direction of relationship and the leading cause of the impact on left-behind children.

## Conclusions

In summary, the findings in this study indicated that compared to non-left-behind children, left-behind children in Sichuan Province were more likely to take sick leave from school with lower coverage of vaccination but they were less likely to experience loneliness. Gender difference is also demonstrated from the result that girls are more vulnerable regarding to certain symptoms and emotional problems. This study provided supportive evidence to inform further reform of household registration system to improve rights of equal access to employment, education and health resources for labor and their children from rural areas in China.

## Additional file

Additional file 1: Survey questionnaire. (DOCX 23 kb)

## Abbreviations

GDP: Gross Domestic Product
USD: United Stated Dollar
