# Supplementary material for: Health status of children left behind in rural areas of Sichuan Province of China: a cross-sectional study
Source: BMC Int Health Hum Rights. 2019 Jan 28;19:4. doi: 10.1186/s12914-019-0191-9 (PMC6350297; doi:10.1186/s12914-019-0191-9)
Supplement: Supplementary file 1 — Survey questionnaire. (DOCX 23 kb) [file 12914_2019_191_MOESM1_ESM.docx]

**Additional file 1: Survey questionnaire**

**Part 1: Background information**

| 1.1 Age: |
| --- |
| 1.2 Gender |
| - Male |
| - Female |
| 1.3 Rural household registration |
| - Yes |
| - No |
| 1.4 Both parents migrating for employment more than 6 months |
| - Yes |
| - No |
| 1.5 Paternal Education |
| - Illiterate |
| - Primary School |
| - Junior High School |
| - High School |
| - College |
| - University or above |
| 1.6 Maternal Education |
| - Illiterate |
| - Primary School |
| - Junior High School |
| - High School |
| - College |
| - University or above |

**Part 2: Physical health**

Please choose the answer according to your own experience.

| 2.1 Occurrence of fever in the past two weeks |
| --- |
| - Yes |
| - No |
| 2.2 Occurrence of cough or respiratory difficulties in the past two weeks |
| - Yes |
| - No |
| 2.3 Occurrence of diarrhea in the past two weeks |
| - Yes |
| - No |
| 2.4 Occurrence of twitch in the past two weeks |
| - Yes |
| - No |
| 2.5 Visit healthcare institute due to sickness in the past two weeks |
| - Yes |
| - No |
| 2.6 Type of healthcare institute visited |
| - Private clinic / private hospital |
| - Village clinic |
| - Township hospitals |
| - County hospitals and above |
| 2.7 Not going to school due to sickness in the past 12 months |
| - Never |
| - 1 time |
| - 2 times |
| - 3 times |
| - 4 times or above |
| 2.8 Take sick leave from school due to sickness in the past 12 months |
| - Did not get sick |
| - Sick but did not take leave |
| - Sick and take leave |
| 2.9 Having the vaccination document |
| - Yes |
| - No |
| - Do not know |
| 2.10 Complete the corresponding vaccination scheme for your age |
| - Yes |
| - No |
| - Do not know |

**Part 3: Mental health**

Please choose the answer according to your own experience in the past 6 months.

| 3.1 Unhappiness due to the study stress or the issues related to the academic result |
| --- |
| - Never |
| - Seldom |
| - Sometimes |
| - Often |
| - Always |
| 3.2 Insomnia due to worrying |
| - Never |
| - Seldom |
| - Sometimes |
| - Often |
| - Always |
| 3.3 Loneliness |
| - Never |
| - Seldom |
| - Sometimes |
| - Often |
| - Always |
| 3.4 Having the idea of running away from home |
| - Never thought |
| - Have thought about it |
| - Tried to run away but failed |
| - Ran away from home |
| 3.5 Stopping the daily activities for 2 consecutive weeks or even longer time due to depression or hopelessness |
| - Yes |
| - No |
